# Post-ERCP biliary complications in patients with biliary type sphincter of Oddi dysfunction

**DOI:** 10.1038/s41598-018-28309-w

**Published:** 2018-07-02

**Authors:** Hiroyuki Miyatani, Hirosato Mashima, Masanari Sekine, Satohiro Matsumoto

**Affiliations:** Department of Gastroenterology, Jichi Medical University, Saitama Medical Center, 1-847 Amanuma, Omiya, Saitama, Saitama, 330-8503 Japan

## Abstract

Sphincter of Oddi dysfunction (SOD) has a high risk of post-ERCP pancreatitis. Cholangitis, colicky pain, and jaundice from cholestasis also occur after ERCP. However, these post-ERCP biliary complications have not been sufficiently evaluated in SOD. Thus, the risk factors and post-ERCP biliary complications in biliary-type SOD were evaluated. From December 1996 to January 2017, 72 patients with suspected biliary-type SOD were selected, and 60 patients who underwent ERCP were included in this study. The incidence of biliary complications compared to control group and factors associated with post-ERCP cholangitis were investigated. More frequent biliary complications, including biliary pain and abnormal liver function, occurred in SOD group than in control group, especially in type I SOD patients. Post-ERCP cholangitis occurred significantly more often with bile duct diameter ≥12 mm (26%, 5/19) than with bile duct diameter <11 mm (2.4%, 1/41; p = 0.016). Age ≥75 years was also a risk factor for post-ERCP cholangitis (p = 0.013). Multivariate analysis confirmed that bile duct diameter ≥12 mm was a significant risk factor for post-ERCP cholangitis. Post-ERCP biliary complications occurred frequently in biliary-type SOD, especially type I. Biliary diameter ≥12 mm was an important risk factor for post-ERCP cholangitis.

## Introduction

Endoscopic retrograde cholangiopancreatography (ERCP) is a well-known invasive procedure with many complications, including pancreatitis. Acute cholangitis is also one of the annoying complications that sometimes occur after ERCP. Unlike post-ERCP pancreatitis, post-ERCP cholangitis is usually preventable by biliary drainage after ERCP. Generally, the frequency of post-ERCP cholangitis is much lower than that of post-ERCP pancreatitis, which is considered to be about 0.7–5%^1–7^. Therefore, it is usually unnecessary to place endoscopic nasobiliary drainage (ENBD) or endoscopic biliary stenting to prevent post-ERCP cholangitis.

Sphincter of Oddi dysfunction (SOD) is a well-known risk factor for post-ERCP pancreatitis. By the Milwaukee classification, biliary-type SOD is classified into 3 types depending on the presence or absence of bile duct dilatation and the presence or absence of liver dysfunction^8,9^. Classical type I SOD is defined by biliary type pain, abnormal liver function tests (LFTs) on two or more occasions, delayed drainage of ERCP contrast >45 min, and a dilated common bile duct (CBD; diameter >12 mm). Post-ERCP cholangitis is suspected to occur easily if ERCP is performed to the bile duct in type I SOD due to delayed drainage of contrast medium. We have sometimes seen biliary complications after ERCP, such as post-ERCP cholangitis and abnormal liver function with or without biliary type pain. Few reports have investigated the incidence and risk factors of post-ERCP cholangitis in SOD patients. Therefore, the question remains of in which biliary-type SOD patients preventive biliary drainage should be placed. To answer this question, the risk factors of post-ERCP cholangitis were investigated, and post-ERCP biliary complications were evaluated in SOD patients.

## Methods

**Figure 1:**
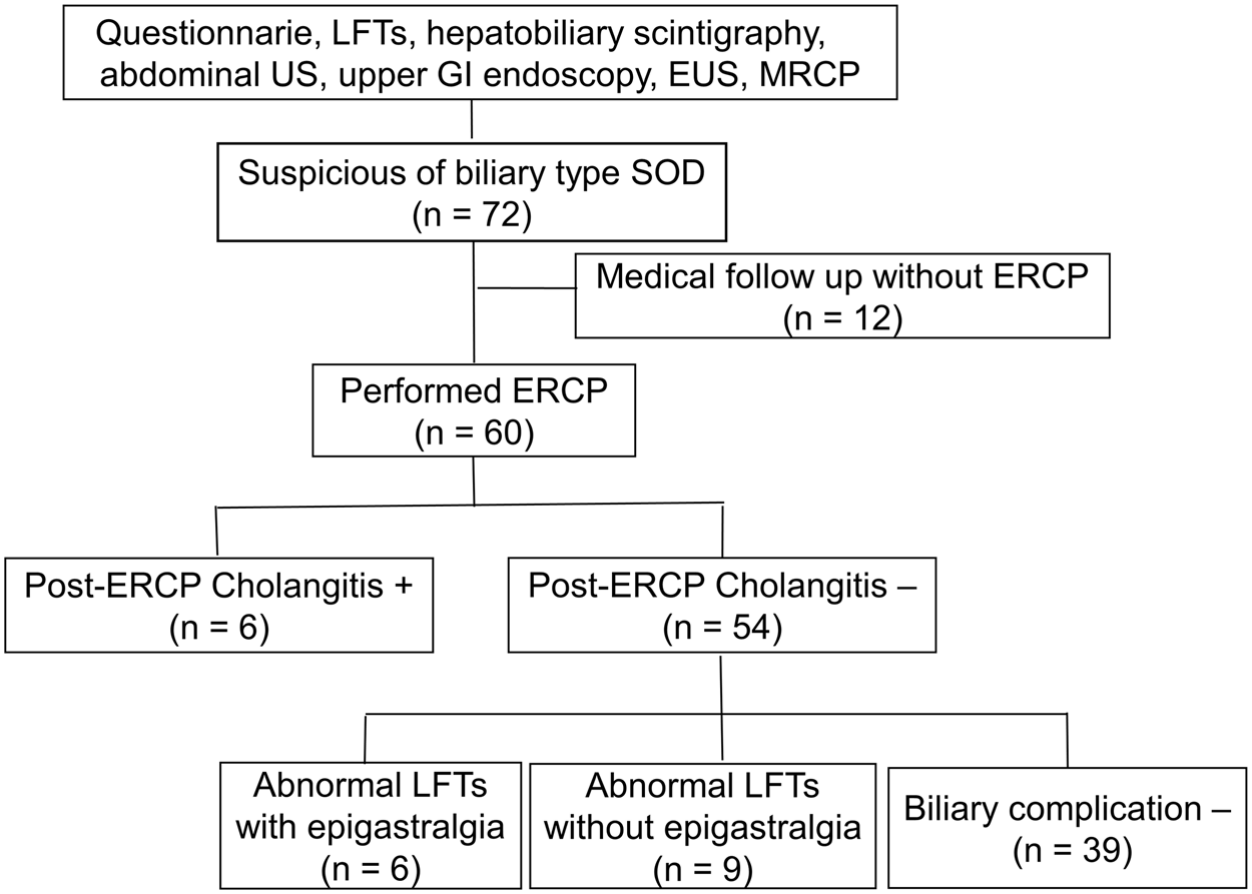
Flow chart of our patient selection and diagnostic strategy.

From December 1996 to January 2017, 72 cases of suspected biliary-type SOD patients were selected by questionnaire, LFTs, hepatobiliary scintigraphy, abdominal ultrasonography, upper gastrointestinal endoscopy, endoscopic ultrasonography, and magnetic resonance cholangiopancreatography. Cases of suspected choledocholithiasis and chronic pancreatitis, obvious mental disorder, previous endoscopic sphincterotomy (EST), and medical treatment (12 cases) were excluded. Sixty patients who underwent ERCP were included in this study (Fig. 1). The registry data of these 60 patients were retrospectively reviewed. Based on clinical, radiographic, and laboratory data, patients were categorized according to the modified Milwaukee classification^9^ as having type I, II, or III SOD. Manometry was performed for type II and III SOD when possible. EST was performed for type I SOD and manometry-confirmed type II and III SOD patients. However, when type I SOD with a low frequency of severe attacks (<2 times/year) was diagnosed, EST was not performed at initial ERCP. After other organic disorders including malignancy and choledocholithiasis were excluded by ERCP, medical treatment was indicated.

As a control group, non-SOD patients performed ERCP (from June 2005 to January 2016, 60 cases) were extracted by matching age and gender as much as possible. Cases of choledocholitiasis, examination for acute cholangitis, malignant or benign biliary stenosis, previously performed EST were excluded. Cholangiography was done in all cases.

It is thought that abnormal LFT with or without abdominal pain after ERCP are caused by a transient increase in biliary pressure. In cholangitis, bacterial growth in the bile duct causes inflammation, requiring antibiotics with or without drainage. To distinguish between them, we used the presence or absence of fever (>38°) as a diagnostic criterion for cholangitis according to the Tokyo guideline 2013^10^. However, there is no diagnostic criteria for cholangitis after ERCP, and it is necessary to consider distinction from pancreatitis, cholecystitis, aspiration pneumonia etc. Post-ERCP cholangitis was defined as fever (>38°) with new or worsened abdominal pain and new or worsened LFTs and requiring treatment with prolonged hospitalization. The severity of post-ERCP cholangitis was graded as mild, moderate, or severe according to Tokyo guideline 2013^10^.

Difficult cannulation was defined on the basis of the time taken for biliary cannulation. An attempt at biliary cannulation lasting longer than 15 minutes was defined as difficult cannulation. When selective bile duct cannulation was difficult, cannulation was attempted using the pancreatic duct guidewire (PGW) technique. In cases of further difficulties, precut sphincterotomy was performed. A prophylactic antibacterial agent was administered on the day of ERCP.

The frequency of biliary complications (post-ERCP cholangitis, new onset or worsened abnormal LFTs with biliary type pain, new onset or worsened abnormal LFTs without biliary type pain) and risk factors associated with post-ERCP cholangitis were investigated in biliary-type SOD. The LFTs were evaluated on the day after ERCP. The incidence of post ERCP cholangitis, bile duct diameter, AST and T-Bil were compared in SOD group and control group.

This study was approved by the Institutional Review Board of our institution (S16–030).

The clinical procedures were carried out in accordance with the Declaration of Helsinki.

All patients signed informed consent related to endoscopic procedures and complications. Regarding this study content, informed consent was not applicable because of the retrospective nature of this study.

### Statistical analysis

The primary outcome analyzed was the development of post-ERCP cholangitis. Univariate analysis was used with Fisher’s exact test for categorical variables. Variables with p values less than 0.15 on univariate analysis were included in step-wise variable selection. Logistic regression analysis for multivariate analysis was used to identify the risk factors for post-ERCP cholangitis. Values of p < 0.05 were regarded as significant. The secondary outcome was the development of other biliary complications. Between-group comparisons were performed using the Mann-Whitney U test, paired t-test and Fisher’s exact test. Values of p < 0.05 were regarded as significant. Statistical analysis was performed using StatFlex version 6.0 (Artech Co., Ltd. Osaka, Japan).

## Results

The basic characteristics of patients with biliary-type SOD group and control group are shown in Table 1. The overall post-ERCP cholangitis rate was 10% (6/60) (mild 3, moderate 3). Three cases of post-ERCP cholangitis occurred on the day after ERCP, 2 occurred two days later, and 1 occurred three days later. Blood cultures were tested in 4 out of 6 cases. No bacteria were detected because we routinely use antibiotics after ERCP. All post-ERCP cholangitis cases were treated with antibiotics without endoscopic biliary drainage. Antibiotics were administered for several days even after antipyretic. On the other hand, post-ERCP cholangitis did not occur in the control group (p < 0.05).

**Table 1:** Basic characteristic of patients with biliary type sphincter of Oddi dysfunction.

	SOD	control	p
No. of patients	60	60	
age of diagnosis (y)	61 ± 15	61 ± 15	
sex (men/women)	19/41	19/41	
previous cholecystectomy	23 (38%)	1 (1.7%)	<0.001
history of pancreatitis	11 (17%)	17 (28%)	0.195
biliary SOD type			
type I	23 (38%)		
type II	28 (47%)		
dilated CBD	5 (8%)		
raised LTTs	23 (39%)		
type III	9 (15%)		
EST	28 (47%)	3 (5%)	<0.001
biliary drainage	4 (7%)	6 (10%)	0.508
manometry	22 (37%)	0 (0%)	<0.001
difficult cannulation	34 (58%)	18 (30%)	0.003
pancreatic duct opacification	42 (71%)	47 (78%)	0.297
precut sphincterotomy	5 (8%)	2 (3%)	0.243
failed cannulation	3 (5%)	0 (0%)	0.242
post ERCP pancreatitis	14 (24%)	8 (13%)	0.157
(mild/moderate/severe)	(6/7/1)	(3/4/1)	
post ERCP cholangitis	6 (10%)	0 (0%)	0.012
(mild/moderate/severe)	(3/3/0)	(0/0/0)	
post ERCP abnormal LFTs (AST > 100 U/l) without cholangitis	15 (25%)	2 (3%)	<0.001
post ERCP abnormal LFTs with epigastralgia	6 (10%)	0 (0%)	0.012
post ERCP abnormal LFTs without epigastralgia	9 (15%)	2 (3%)	0.027
biliary diameter, mm (mean ± SD)	11 ± 5	7.7 ± 2.8	<0.001
AST, U/l (mean ± SD)	112 ± 167	31 ± 38	<0.001
T-Bil, mg/dl (mean ± SD)	1.3 ± 0.9	0.9 ± 0.5	0.011

*SOD* sphincter of Oddi dysfunction, *CBD* common bile duct, *LFTs* liver function tests, *EST* endoscopic sphincterotomy, *AST* aspartate aminotransferase.

No procedure-related deaths and no hospital deaths occurred in any of the patients with complications.

The data were analyzed using a logistic regression model with 13 potential risk factors for post-ERCP cholangitis. Univariate analysis showed that age ≥75 years and bile duct diameter ≥12 mm were significant risk factors for post-ERCP cholangitis (Table 2).

**Table 2:** Risk factors for post-ERCP cholangitis in biliary type sphincter of Oddi dysfunction patients (univariate analysis).

	Cholangitis			
	(+) (*n* = 6)	(−) (*n* = 54)	p	OR (95% CI)
age ≥ 75 (y)	4	8	0.013	11.50 (1.78~73.58)
male gender	1	18	0.711	0.40 (0.04~3.68)
previous cholecystectomy	1	22	0.478	0.29 (0.03~2.66)
biliary SOD type				
type I	5	18	0.051	10.00 (1.09~92.11)
type II + III	1	36	0.051	0.10 (0.01~0.92)
manometry	3	19	0.789	1.84 (0.34~10.03)
bile duct diameter ≥12 (mm)	5	14	0.016	14.29 (1.53~133.08)
abnormal LFTs	5	41	0.919	1.59 (0.17~14.83)
difficult cannulation	2	25	0.565	1.44 (0.42~4.98)
EST	5	23	0.142	6.74(0.74~61.67)
biliary drainage	0	4	0.863	0.00 (1.00)
precut sphincterotomy	0	5	1	0.00 (1.00)
post-ERCP pancreatitis	3	3	0.263	3.91 (0.69~22.09)

*SOD* sphincter of Oddi dysfunction, *LFTs* liver function tests, *EST* endoscopic sphincterotomy.

Though not significant, post-ERCP cholangitis tended to occur in type I SOD patients, but not in type II/III SOD patients. Biliary SOD type I is closely related to bile duct dilatation, and it was excluded from multivariate analysis due to suspected multiple collinearity. Among them, only bile duct diameter ≥12 mm was confirmed to be a risk factor for post-ERCP cholangitis on multivariate analysis (Table 3).

**Table 3:** Risk factors for post-ERCP cholangitis in biliary type sphincter of Oddi dysfunction patients (multivariate analysis).

	*p*	OR (95% CI)
age ≥ 75 (y)	0.089	6.26 (0.75~52.09)
bile duct diameter ≥12 (mm)	0.041	11.80 (1.11~125.33)
EST	0.278	3.93 (0.33~46.79)

*EST* endoscopic sphincterotomy.

In the group without post-ERCP cholangitis, the rate of post-ERCP abnormal LFTs (AST > 100 U/L) with epigastralgia and without epigastralgia was 10% (6/60) and 15% (9/60), respectively. Post-ERCP cholangitis occurred in 21.7% (5/23) of biliary SOD type I cases (Table 4). Total biliary complications including cholangitis were seen in 52.2% (12/23) of type I SOD patients, which was higher than the 24.3% (9/37) seen in type II + III SOD patients (p < 0.05). In control group, only 2 cases occurred post ERCP abnormal LFTs without epigastralgia.

**Table 4:** Features of post-ERCP biliary complications.

	cholangitis (*n* = 6)	abnormal LFTs epigastralgia + (*n* = 6)	abnormal LFTs epigastralgia - (*n* = 9)	no biliary complication (*n* = 39)
biliary diameter, mm (mean ± SD)	17.7 ± 7.8	11.4 ± 4.4	11.6 ± 3.9	9.4 ± 3.7
SOD type I	5 (21.7%)	1 (4.3%)	6 (26.1%)	11 (47.8%)
II	0	5 (17.9%)	3 (10.7%)	20 (71.4%)
III	1 (11.1%)	0	0	8 (88.9%)
AST, U/l (mean ± SD)	276 ± 329	171 ± 69	230 ± 238	47 ± 52
T-Bil, mg/dl (mean ± SD)	2.2 ± 1.2	2.9 ± 1.4	1.1 ± 0.4	0.9 ± 0.4

*LFTs* liver function tests, *SOD* sphincter of Oddi dysfunction, *AST* aspartate aminotransferase, *T-Bil* total bilirubin.

Biliary diameter was significantly larger in post-ERCP cholangitis cases than in the other biliary complication group and the no complication group (p < 0.01). The serum aspartate aminotransferase (AST) level one day after ERCP was higher in the biliary complication groups than in the no complication group (p < 0.01). The serum total bilirubin (T-Bil) level one day after ERCP was higher in the post-ERCP cholangitis and abnormal LFTs with epigastralgia group than in the abnormal LFTs without epigastralgia group and the no biliary complication group (p < 0.05). In control group, biliary diameter was significantly smaller than in SOD group and serum AST and T-Bil level were also significantly lower than in SOD group.”

## Discussion

Post-ERCP pancreatitis is a very debilitating complication of ERCP. There is no established method for reliable prevention of post-ERCP pancreatitis. Other complications including perforation, bleeding at endoscopic papillotomy, and cholangitis are also known to occur with ERCP. The frequency of post-ERCP cholangitis is relatively less than that of post-ERCP pancreatitis, which is considered to be about 0.7–5%^1–7^. However, once post-ERCP cholangitis occurs, medical and/or endoscopic treatment is needed. There are reports that the mortality rate of post-ERCP cholangitis is 0.3–0.9%^4,6^. The major point where post-ERCP cholangitis differs from post-ERCP pancreatitis is that prevention is possible to some extent. ENBD reduces the incidence of cholangitis in patients with EST and repeated stone extraction^10^. The frequency of post-ERCP cholangitis was reduced by antibiotic prophylaxis^5,11^. Although ENBD or temporary biliary stenting can prevent post-ERCP cholangitis, drainage is not always necessary for all biliary-type SOD cases. The present study result showed that post-ERCP cholangitis is likely to occur with a large common bile duct (CBD) diameter. In the previous report, the risk factors for post-ERCP cholangitis were increased CBD diameter, biliary dilatation, biliary stent insertion, prolonged total procedure time, and hilar cholangiocarcinoma^4^. In addition, prior cholecystectomy, small center, cholestasis, and bile duct malignancy are considered risk factors for post-ERCP cholangitis^7,12^. Although biliary stenting is considered to prevent post-ERCP cholangitis to some extent, it was controversially reported to be a risk factor for post-ERCP cholangitis. This may be due to stent dislocation and/or acute obstruction by viscous bile. In this respect, ENBD seems to be more reliable than biliary stenting because we can monitor the amount of bile juice. The previous report on endoscopic drainage for acute suppurative cholangitis showed that ENBD and plastic stenting were equally effective and safe^13,14^. A prospective study with a large number of cases is needed to confirm whether ENBD or biliary stenting can prevent post-ERCP cholangitis in biliary-type SOD patients.

SOD, especially type I, is thought to represent a relative cholestasis state. A large-diameter CBD was a risk factor for post-ERCP cholangitis in the present study. Inadequate biliary excretion from a stenotic duodenal papilla after the procedure may cause cholangitis. EST is expected to prevent post-ERCP cholangitis by promotion of biliary excretion, but the present results did not show an effect, or rather it might contribute to post-ERCP cholangitis with no significant difference. This may be due to stronger papillary edema occurring after EST in SOD cases than in other cases. Cautery-induced papillary edema occurs more often in high-risk patients, such as SOD patients, and is one of the causes of pancreatitis^15^. This mechanism can also explain post-ERCP cholangitis. We usually do not place an ENBD tube or tube stent after EST in SOD patients. As an exception, we recently placed an ENBD tube in a type I SOD case to avoid cholangitis based on the result of the present study, and the patient’s clinical course was uneventful.

In the present study, there were many complications after ERCP for SOD patients, such as biliary pain and liver abnormalities without cholangitis. These complications after ERCP for SOD patients have not been reported, and this may be due to much attention being paid to post-ERCP pancreatitis. Biliary complications including cholangitis occurred more frequently in type I SOD than in type II and type III SOD. Papillary stenosis in SOD may cause acute cholestasis, followed by biliary pain or liver injury, resulting in cholangitis due to bacterial infection^16–18^.

This study has some limitations. It was a retrospective, single-center study. For that reason, the number of cases was relatively small. Therefore, a large multicenter study is needed to confirm the incidence and risk factors of post-ERCP cholangitis in biliary-type SOD. Such a study will confirm whether temporary biliary drainage is effective for high-risk patients.

In conclusion, post-ERCP biliary complications occurred frequently in biliary-type SOD patients. Biliary diameter ≥12 mm was an important risk factor for post-ERCP cholangitis. After ERCP, patients must be carefully observed for the development of fever and abdominal pain, especially type I SOD patients.
